# Phase engineering of layered anode materials during ion-intercalation in Van der Waal heterostructures

**DOI:** 10.1038/s41598-023-31342-z

**Published:** 2023-04-03

**Authors:** Shayani Parida, Arthur Dobley, C. Barry Carter, Avinash M. Dongare

**Affiliations:** 1 Department of Materials Science and Engineering, University of Connecticut, Storrs, CT USA; 2 EaglePitcher Technologies, Providence, RI USA; 3 Department of Chemical and Biomolecular Engineering, University of Connecticut, Storrs, CT USA; 4 Center for Integrated Nanotechnologies (CINT), Sandia National Laboratories, Albuquerque, NM USA

**Keywords:** Nanoscale materials, Theory and computation

## Abstract

Transition metal dichalcogenides (TMDs) are a class of 2D materials demonstrating promising properties, such as high capacities and cycling stabilities, making them strong candidates to replace graphitic anodes in lithium-ion batteries. However, certain TMDs, for instance, MoS_2_, undergo a phase transformation from 2H to 1T during intercalation that can affect the mobility of the intercalating ions, the anode voltage, and the reversible capacity. In contrast, select TMDs, for instance, NbS_2_ and VS_2_, resist this type of phase transformation during Li-ion intercalation. This manuscript uses density functional theory simulations to investigate the phase transformation of TMD heterostructures during Li-, Na-, and K-ion intercalation. The simulations suggest that while stacking MoS_2_ layers with NbS_2_ layers is unable to limit this 2H → 1T transformation in MoS_2_ during Li-ion intercalation, the interfaces effectively stabilize the 2H phase of MoS_2_ during Na- and K-ion intercalation. However, stacking MoS_2_ layers with VS_2_ is able to suppress the 2H → 1T transformation of MoS_2_ during the intercalation of Li, Na, and K-ions. The creation of TMD heterostructures by stacking MoS_2_ with layers of non-transforming TMDs also renders theoretical capacities and electrical conductivities that are higher than that of bulk MoS_2_.

## Introduction

Lithium-ion batteries (LIBs) are rechargeable energy storage devices that are rapidly replacing nickel–cadmium and nickel metal hybrid batteries^1,2^. LIBs’ main benefits over their counterparts include their high energy density, no memory effect, and low self-discharge^3–5^. However, LIBs present their own production and performance challenges, including, but not limited to, the scarcity of Li and its expensive mining process and limited theoretical capacity of 372 mAh/g for graphitic anodes^6,7^. Thus, current efforts seek to identify intercalating species that could substitute Li in the next generation batteries as well as discover/design anode materials beyond graphite. Potential candidates for intercalating species include other monovalent metals, such as Na^8,9^ and K^10,11^, and multivalent metals, such as Mg^12–15^, Ca^12,15,16^, and Al^12,15,17^. Similarly, potential candidates for anodes are transition metal dichalcogenides (TMDs) owing to their high capacities and large interlayer spacings for facile movement of intercalating ions^18–20^.

Understanding the behavior of TMDs at the atomic level is crucial to predict their structural stability during charge–discharge cycles when they are used as anodes in batteries. TMDs, including MoS_2_, can exhibit multiple phases depending on the coordination of the chalcogen atoms around the metal atoms, primarily the 2H (where the metal lies at the center of a trigonal prismatic coordination sphere) and 1T (where the metal lies at the center of an octahedral coordination sphere) phases. Upon intercalation of Li-ions, MoS_2_ transforms from 2H to 1T phase^21,22^. On the other hand, such a phase transformation does not occur in certain TMDs, such as NbS_2_ and VS_2_^23,24^. The intercalation of metal ions in MoS_2_ layers leads to a transfer of electrons from the ion to MoS_2_. The excess negative charge around Mo leads to a change of the coordination state around the Mo atom, resulting in a transition from the 2H to 1T phase^25^. On the other hand, experiments have shown that the hexagonal 2H phase of NbS_2_ is stable at high Li concentrations during cell cycling^26,27^. The 2H phase of NbS_2_ is observed to be more stable than the 1T phase upon gaining electrons. Thus, electropositive ions, like Li, Na, K, etc., which donate electrons upon intercalation, do not cause phase transformation in NbS_2_. On the contrary, phase transformation of NbS_2_ has been proposed to be possible upon intercalation of electron-accepting impurities^23^.

When metal ions intercalate into the bulk of MoS_2_, in-situ TEM characterization suggests that the ion concentration is not uniform throughout the lattice. This leads to the formation of packets of lithiated regions with varying concentrations within the lattice^28–30^. Both experimental and computational studies have suggested that the ion-intercalated regions tend to phase transform to 1T while the non-intercalated regions remain in the 2H phase^22,31,32^. The two phases exhibit different ion mobilities, and their phase boundaries impede ion diffusion^33^. Additionally, the interface between them experiences large stresses due to volumetric differences between the phases, and this can lead to mechanical failure^34^. Thus, phase engineering, specifically the stabilization of the phases, is imperative for the improvement of battery performance.

Van der Waal (vdW) heterostructures of 2D materials promise interesting new properties which can be significantly different from the average of the properties of the individual layers^35–37^. Unlike the creation of in-plane interfaces, the formation of interfaces in vdW heterostructures leads to clean interface structures since bond breaking or formation does not occur. vdW heterostructures have been explored for different applications^38–40^. Zhong et al. created vdW heterostructures of ferromagnetic semiconductor CrI_3_ and a TMD, WSe_2,_ and observed significant changes in spin and valley pseudospin in the TMD^41^. Xue et al. observed Moiré patterns in the heterostructures of graphene and hexagonal boron nitride^42^. In the context of batteries, Peng et al. demonstrated the use of vertical heterostructures of blue phosphorene and MS_2_ (M = Nb, Ta) as flexible anode materials with capacities reaching 530 mAh/g^43^. The vdW heterostructures of bimetallic oxychloride and reduced graphene oxides have been proposed to be suitable candidates for potassium-battery anodes^44^. MoS_2_ and doped graphene heterostructures have also proven to be efficient anodes for LIBs^45^. There are certain avenues for the application of vdW heterostructures that are still not well understood. For instance, the synergy between stacked 2D layers can also influence the binding of intercalating ions and can potentially be exploited to regulate localized phase-transition behavior in thin films of 2D heterostructures.

This computational study investigates the phase stability of model vdW heterostructures created by stacking layered TMDs during ion intercalation. The vdW heterostructures stack layered TMDs that phase transform with those that do not phase transform to create vertical interfaces. These vertical interfaces will affect the binding of the intercalating ions and, hence, the phase stability of the layers. Density functional theory (DFT) simulations investigate the phase transformation of TMD heterostructures during Li-, Na-, and K-ion intercalation. MoS_2_ is used here to model the transforming TMD layer, while NbS_2_ and VS_2_ represent the non-phase transforming TMD layers. The study suggests that introducing a layer of NbS_2_ or VS_2_ between layers of MoS_2_ could potentially impede the 2H to 1T phase transformation of MoS_2_ layers during Na or K intercalation. The differences in phase transformation behavior are due to the differences in charge transfer between the intercalating ion and the TMD layers. This study investigates the role of the interactions between the intercalating ions and the TMD layers on the phase stability of vdW heterostructures. In addition, the study identifies that the distribution of vertical interfaces also plays a role in impeding the phase transformation behavior during intercalation. This study identifies that introducing one non-transforming layer can impede the phase transformation behavior of only a limited number of MoS_2_ layers in the vdW heterostructure. In addition, these vdW heterostructures show improved theoretical capacity and electrical conductivity.

## Results and discussion

### Phase transformation in bulk MoS_2_, NbS_2,_ and VS_2_ in the presence of various intercalating ions

**Figure 1:**
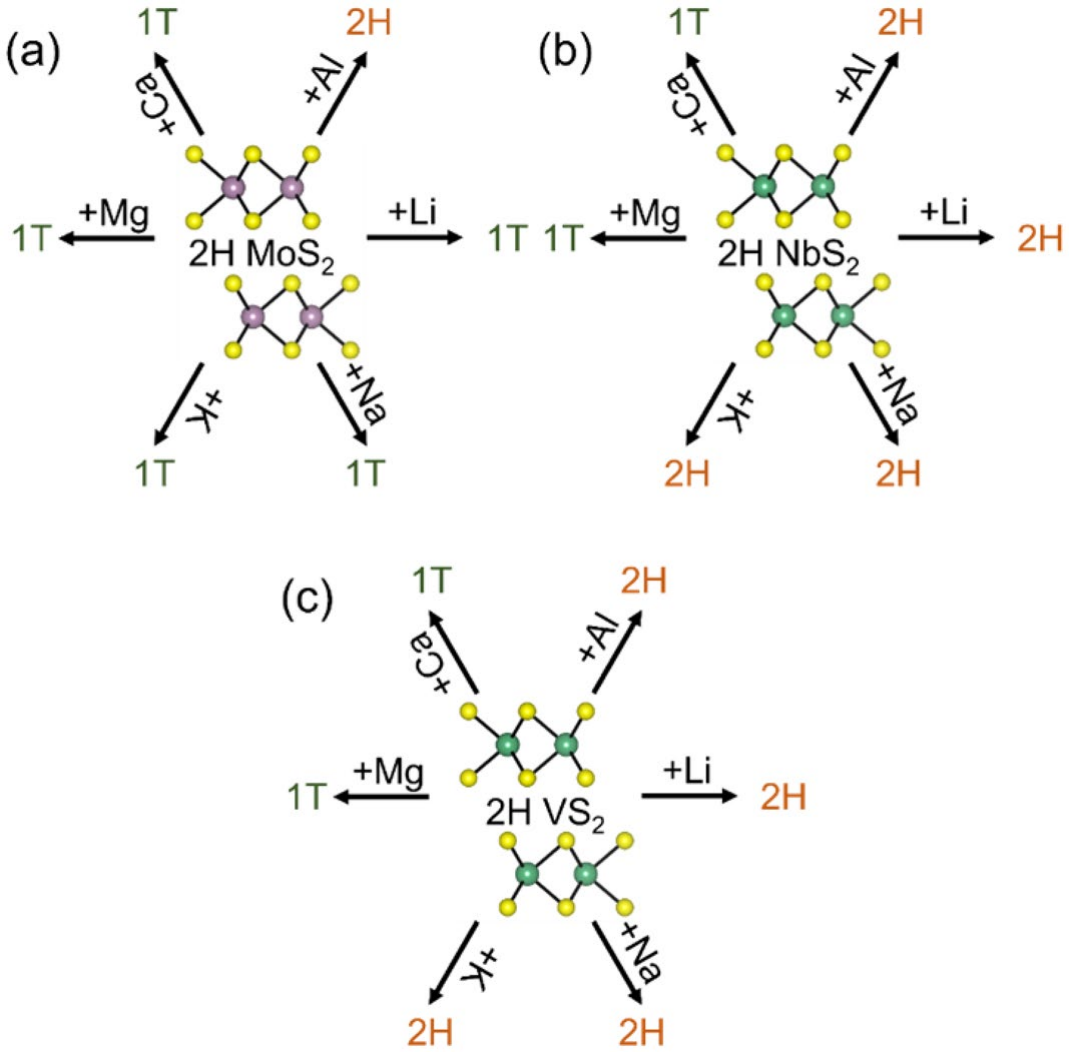
Schematic showing the resultant phases upon intercalation of various ions in 2H-(**a**) MoS_2_, (**b**) NbS_2_ and (**c**) VS_2_.

As the first step, DFT simulations are carried out to determine the energetics of the TMD phases in the presence of Li and other intercalating ions, Na, K, Mg, Ca, and Al. Multilayered TMDs have two high symmetry sites where Li typically prefers to bind, a sixfold coordinated octahedral site (O_h_) site and a fourfold coordinated tetrahedral (T_d_) site. DFT calculations investigate the energetics of the three TMDs (MoS_2,_ NbS_2,_ and VS_2_) in the intercalated configurations for each binding site. The energetics of the TMDs for each binding site for each intercalating ion is tabulated in Note 1 of Supplemental Information. The energies of the three TMDs in 2H and 1T phases, upon intercalation with these ions at the lowest energy sites are summarized in Table 1 and illustrated in Fig. 1. From the table, it can be concluded that intercalation of Li-, Na-, and K-ions tend to cause the transformation of MoS_2_ from 2H to 1T phase, but intercalated NbS_2_ and VS_2_ remain in the 2H phase. Even when all the Octahedral (O_h_) sites in NbS_2_ or VS_2_ are lithiated, the energy of 2H phase is lower than 1T phase. The results are in accordance with other studies which showed that MoS_2_^46^ transforms from 2H to 1T phase upon lithiation while NbS_2_^46^ and VS_2_^47,48^ resist transformation upon lithiation. All three TMDs tend to phase transform from 2H to 1T phase in the presence of Ca and Mg, while none of them transform when Al is intercalated. This suggests that Group-I alkali metals (Li, Na, and K) lead to a phase transformation of MoS_2_ but not for NbS_2_ or VS_2_. In addition, Group-II alkaline earth metals (Mg and Ca) lead to phase transformation of all the TMDs, and the chosen Group-III metal, Al, triggers phase transformation in none of the TMDs. Such differences in phase transformation behavior can stem from the extent of the interaction between TMDs and intercalating ions. Interesting synergistic effects can be expected during ion intercalation in heterostructures where one of the layered materials transforms while the other does not. Heterostructures of (a) NbS_2_ and MoS_2_ and (b) VS_2_ and MoS_2_ in the presence of Li, Na, and K satisfy this requirement and, therefore, are explored in this study.

**Table 1:** The calculated energy of 2H and 1T phases of ion intercalated MoS_2_, VS_2_, and NbS_2_.

Ion	MoS_2_ (eV/formula unit)		NbS_2_ (eV/formula unit)		VS_2_ (eV/formula unit)	
	2H	1T	2H	1T	2H	1T
–			**− 22.61**	− 22.55	**− 20.41**	− 20.40
Li	− 25.83	**− 26.06**	**− 27.05**	− 26.61	**− 24.78**	− 24.50
Na	− 24.95	**− 25.03**	**− 26.14**	− 25.59	**− 23.96**	− 23.57
K	− 24.30	**− 24.39**	**− 25.64**	− 25.05	**− 23.25**	− 22.96
Mg	− 24.59	**− 25.05**	− 25.45	**− 25.61**	− 23.13	**− 23.47**
Ca	− 25.98	**− 26.50**	− 26.91	**− 27.08**	− 24.56	**− 24.89**
Al	**− 26.38**	− 26.05	**− 26.58**	− 26.40	**− 24.48**	− 24.26

The numbers in bold identify the lower energy phases of the TMDs in the presence of various intercalating ions.

### Intercalation of MoS_2_/NbS_2_ heterostructures with Li-ions

TMD heterostructures are first created by stacking one layer of MoS_2_ on top of one layer of NbS_2_ to understand the effect of interfaces on the phase transformation behavior of MoS_2_ and NbS_2_. This creates a heterostructure of alternating MoS_2_ and NbS_2_ layers and is referred to as the 1-MoS_2_/1-VS_2_ structure, as shown in Figure S1 of the Supplementary Information. Heterostructure configurations are created to test the phase stability of the MoS_2_ and the NbS_2_ layers. The DFT computed energies of different phase combinations in the MoS_2_/NbS_2_ heterostructures are tabulated in Note 2 (Table S2A) of the Supplemental Information that suggests that the heterostructure with alternating NbS_2_ and MoS_2_ layers exist in the 2H phase.

**Figure 2:**
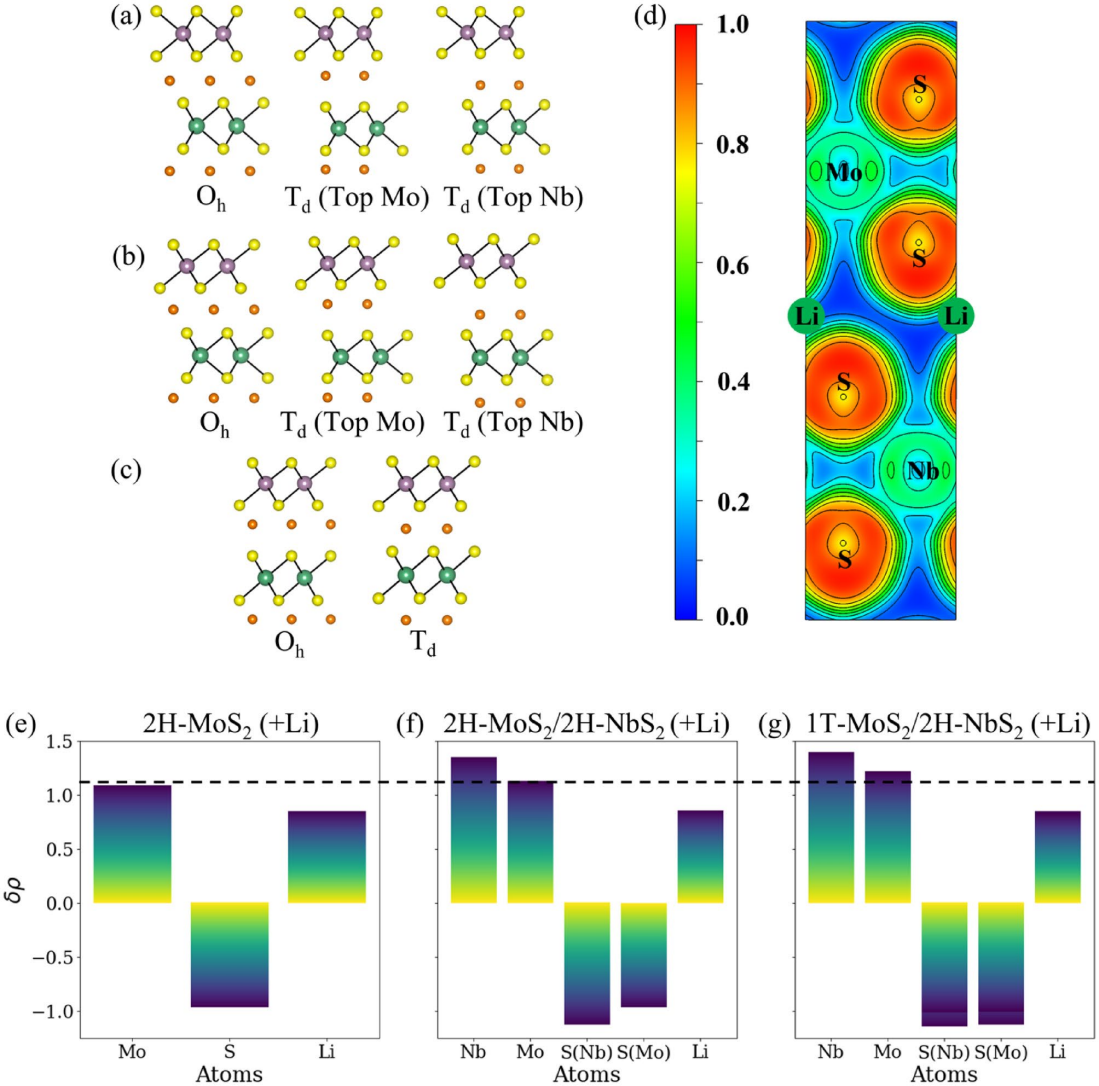
Different high symmetry binding sites considered for Li adsorption are shown in (**a**) 2H-MoS_2_/2H-NbS_2_, (**b**) 1T-MoS_2_/2H-NbS_2_, and (**c**) 1T-MoS_2_/1T-NbS_2_ heterostructures. Purple, green, yellow, and orange spheres denote Mo, Nb, S and Li atoms respectively. (**d**) Electron localization function (ELF) projected on the [100] plane of lithiated 2H-MoS_2_/2H-NbS_2_. A positive value implies that the atom lost electrons, while a negative value indicates that the atom has gained electrons. The dotted line, corresponding to the charge on Mo-atom in lithiated 2H-MoS_2_/2H-NbS_2_, is added to compare the electronic charge on Mo-atom.

DFT calculations are carried out for configurations of lithiated MoS_2_/NbS_2_ heterostructures with Li-ions at various high-symmetry sites to investigate the phase energetics. While multilayered TMDs only have two high-symmetry ion adsorption sites, additional high-symmetry configurations are likely in heterostructures where different TMD layers are stacked. Figure 2a–c illustrates these possible sites. While the H site has O_h_ symmetry, and TopMo and TopNb sites have T_d_ symmetry in the 2H-MoS_2_/2H-NbS_2_ heterostructure, as shown in Fig. 2a, the opposite is true for the 1T-MoS_2_/2H-NbS_2_ heterostructure. However, TopNb and TopMo represent the same site in the 1T-MoS_2_/1T-NbS_2_ heterostructure, as shown in Fig. 2c. Note 2 (Table S2B) of the Supplemental Information compares the energies of various lithiated phases for the possible Li binding sites. The values suggest that the energies of the lithiated heterostructure are very similar for the MoS_2_ layer in the 2H and 1T phases, 1T phase slightly favored by 0.01 eV/formula unit. The previous study^22^ suggested that lithiation of MoS_2_ layers favors a 2H $\to 1\mathrm{T}$ phase transformation, with the 1T phase being thermodynamically more stable than the 2H phase by 0.24 eV. Thus, introducing an NbS_2_ layer between two layers of MoS_2_ can significantly reduce the difference in energy between the 2H and 1T phases of MoS_2_.

A possible reason for the NbS_2_ layer stabilizing the 2H phase in lithiated-MoS_2_/NbS_2_ heterostructures is the stronger binding of Li to NbS_2_. The DFT calculated binding energy (BE) of Li is -2.54 eV in 2H-NbS_2_ and -0.86 eV in 2H-MoS_2_. This difference in Li-ion binding results in the adsorption distances between Li—NbS_2_ and Li—MoS_2_. The distance between the Li-ions and NbS_2_ layer is calculated to be 2.45 Å and is smaller than the distance of 2.56 Å between the ions and MoS_2_ layers in the heterostructure. The origins of the differences in binding can be explored by quantifying the charge transferred between 2D material and the intercalating ions. Figure 2d shows the electron localization around various atoms in the lithiated MoS_2_/NbS_2_ heterostructure. The contour lines show a slight difference in the localization of electron gas around the S atom attached to the Mo atom and the S atom attached to the Nb atom. Figure 2e–g shows the charge transfer between Li-ions and multilayered 2H-MoS_2_ quantified using Bader charge analysis^49^. Nb is more electropositive than Mo, which would result in comparatively more electron transfer from Nb to bonded S atoms than Mo to S atoms. This difference can be clearly seen in Fig. 2f, g. Thus, S attached to Nb would have a greater electronic charge than S bonded to Mo. Hence, positively charged Li would form a stronger ionic bond with S attached to Nb than S bonded to Mo atom. The weaker interaction between Li and S attached to Mo would also result in Mo atoms not gaining enough electron charge during lithiation. Comparing the charges on the Mo atoms in multilayered MoS_2_ as shown in (Fig. 2e) vs in the 2H-MoS_2_/2H-NbS_2_ heterostructure (Fig. 2f), Mo atoms gain more electron charges in bulk than in the heterostructure (the difference being 0.05 e^-^/Mo). The net negative charge on MoS_2_ is the sum of charges on Mo and the two attached S atoms. In the same heterostructure, the net negative charge on MoS_2_ is higher when it exists in the 1T (Fig. 2g) phase than in the 2H phase (Fig. 2f). The barrier for phase transformation of MoS_2_ in the heterostructure in the presence of Li is calculated to be 0.85 eV, which is lower than the transition barrier of 0.95 eV in multilayered MoS_2_. So, kinetically, phase transformation of the lithiated heterostructure is also slightly more favorable than a multilayered MoS_2_ system. The Bader charges on partially phase-transformed MoS_2_ at the saddle point in bulk MoS_2_ and the heterostructure are − 0.88 e^-^ and − 0.82 e^-^ respectively. This is again consistent with the fact that the presence of NbS_2_ depletes the net negative charge on MoS_2_ during Li intercalation.

### Intercalation of MoS_2_/NbS_2_ heterostructures with Na- and K-ions

DFT calculations are also carried out for various configurations of MoS_2_/NbS_2_ heterostructures intercalated with Na- and K-ions at different high-symmetry sites to investigate the phase energetics. Note 2 (Table S2B) of the Supplemental Information compares the energies of various phases of the MoS_2_ and NbS_2_ layers in the intercalated heterostructure for the possible Na-ion and K-ion binding sites. The lowest energy phases of the intercalated heterostructures and the corresponding energies are tabulated in Table 2 for the Na-ion intercalation and K-ion intercalation in addition to that observed for Li-ion intercalation. It can be seen that the intercalated 2H-MoS_2_/2H-NbS_2_ heterostructure has minimum energy, wherein the H site (with O_h_ symmetry) is the energetically preferred binding site. Thus, Na-ion or K-ion intercalation allows the 2H phase of MoS_2_ to be stable.

**Table 2:** Energy of different phases of lithiated 1-MoS_2_/1-NbS_2_ heterostructure (with alternating layers of MoS_2_ and NbS_2_) after Li, Na and K intercalation.

Ion	2H-MoS_2_/2H-NbS_2_ (eV/formula unit)	1T-MoS_2_/2H-NbS_2_ (eV/formula unit)	1T-MoS_2_/1T-NbS_2_ (eV/formula unit)
Li	− 52.75	**− 52.76**	− 52.64
Na	**− 50.96**	− 50.66	− 50.56
K	**− 49.93**	− 49.49	− 49.43

The numbers in bold identify the lower energy phases of the TMDs in the presence of various intercalating ions.

**Figure 3:**
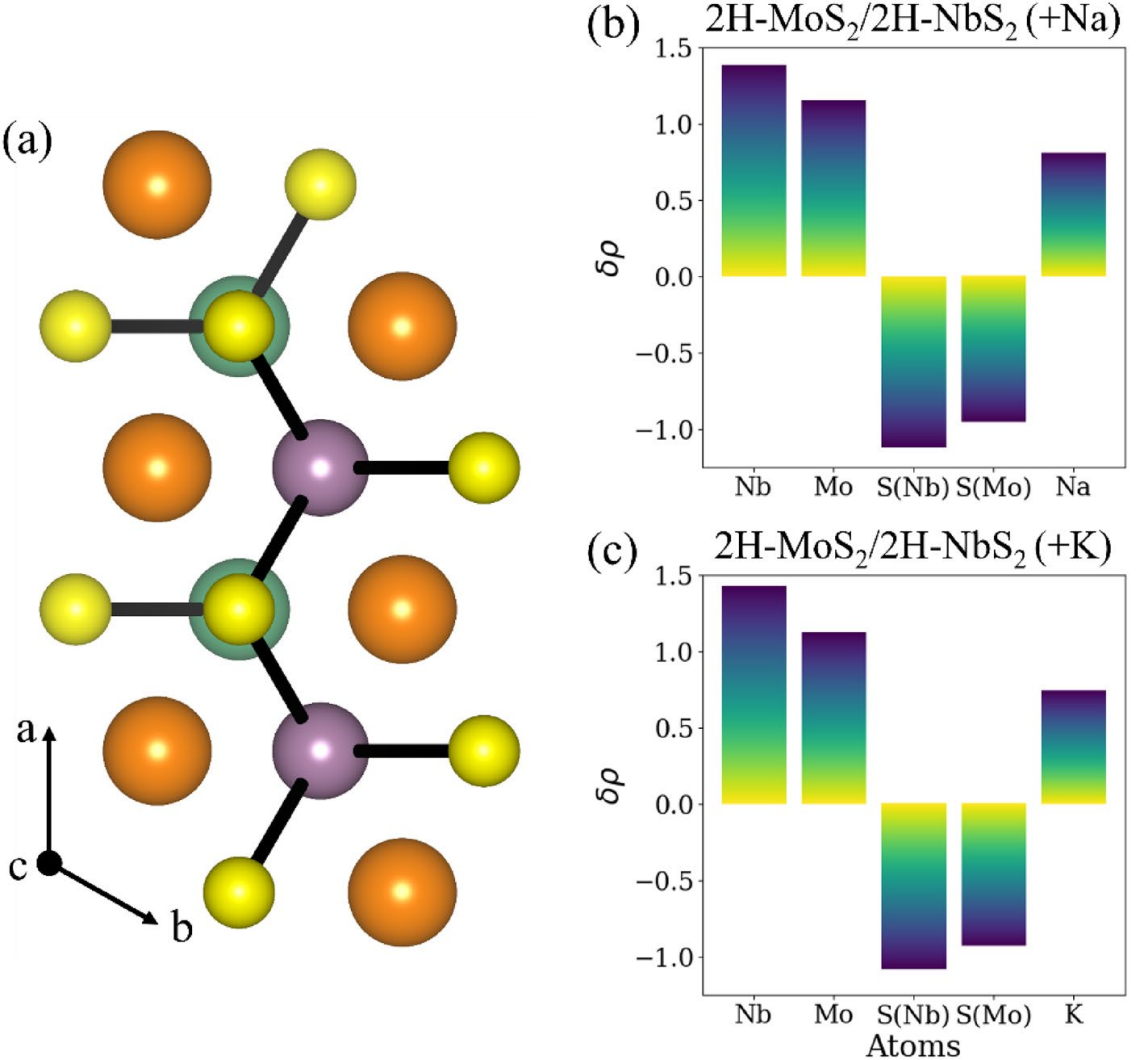
(**a**) Top view of 2H-MoS_2_/2H-NbS_2_ heterostructures (alternating layers of NbS_2_ and MoS_2_) with ions at the lowest energy binding site (O_h_/H site). Charges transferred from different atoms in (**b**) Na-ion intercalated and (**c**) K-ion intercalated heterostructures are computed using Bader analysis. A positive value implies that the atom lost electrons, while a negative value indicates that the atom has gained electrons.

The top view of the intercalated heterostructure is shown in Fig. 3a. Bader analysis is used to analyze the electron transfer in the Na-ion and K-ion intercalated heterostructure, as shown in Fig. 3b,c. It can be concluded that S atoms attached to Nb gain more negative charges than the S atoms attached to the Mo atoms. This difference in charge transfer pulls the positively charged ions toward the NbS_2_ layer. For Na-ion intercalation, the distance between the Na-ions and NbS_2_ layer is calculated to be 2.73 Å and is smaller than the distance of 2.81 Å between the Na-ions and MoS_2_ layers in the heterostructure. For K-ion intercalation, the distance between the K-ions and NbS_2_ layer is calculated to be 3.04 Å and is smaller than the distance of 3.06 Å between the K-ions and MoS_2_ layers in the heterostructure. This would again result in lesser interaction between intercalating ions and MoS_2_ layers and a lesser tendency for MoS_2_ to phase transform.

Thus, the key difference that explains the phase transformation of MoS_2_ in the heterostructure observed during Li-ion intercalation and not during Na-ion or K-ion intercalation lies in the net negative charge on MoS_2_ upon ion intercalation. The net negative charge on MoS_2_ is -0.76 e^-^ for Na-ion intercalation and -0.73 e^-^ upon K-ion intercalation and is lower than the net negative charge of 0.80 e^-^ for Li intercalation. A lower new negative charge on MoS_2_ impedes its phase transformation during Na-ion, and K-ion intercalation, as observed for Li-ion intercalation. The calculated barriers for phase transformation of a MoS_2_ layer in the heterostructures are 0.9 eV and 0.86 eV during Na-ion and K-ion intercalation, respectively. The presence of an interface increases these transformation barriers to 1.05 eV and 0.79 eV during Na-ion and K-ion intercalation, respectively. In the heterostructure, the charges on MoS_2_ at the saddle point during Na and K intercalation are − 0.79 e^-^ and − 0.72 e^-^.

Thus, NbS_2_ exhibits stronger binding with Li-, Na-, and K-ions than MoS_2,_ which weakens the interaction between ions and MoS_2_ during intercalation. However, it is unclear if the introduction of an NbS_2_ impedes phase transformation in only the neighboring MoS_2_ layers or if it can also influence the transformation behavior of non-adjacent MoS_2_ layers. The study, therefore, explores the capability of each NbS_2_ layer to limit to impede the phase transformation of non-adjacent MoS_2_ layers in multilayered heterostructures.

### Intercalation of MoS_2_/NbS_2_ multilayered heterostructures with more than one MoS_2_ layer between two NbS_2_ layers

**Figure 4:**
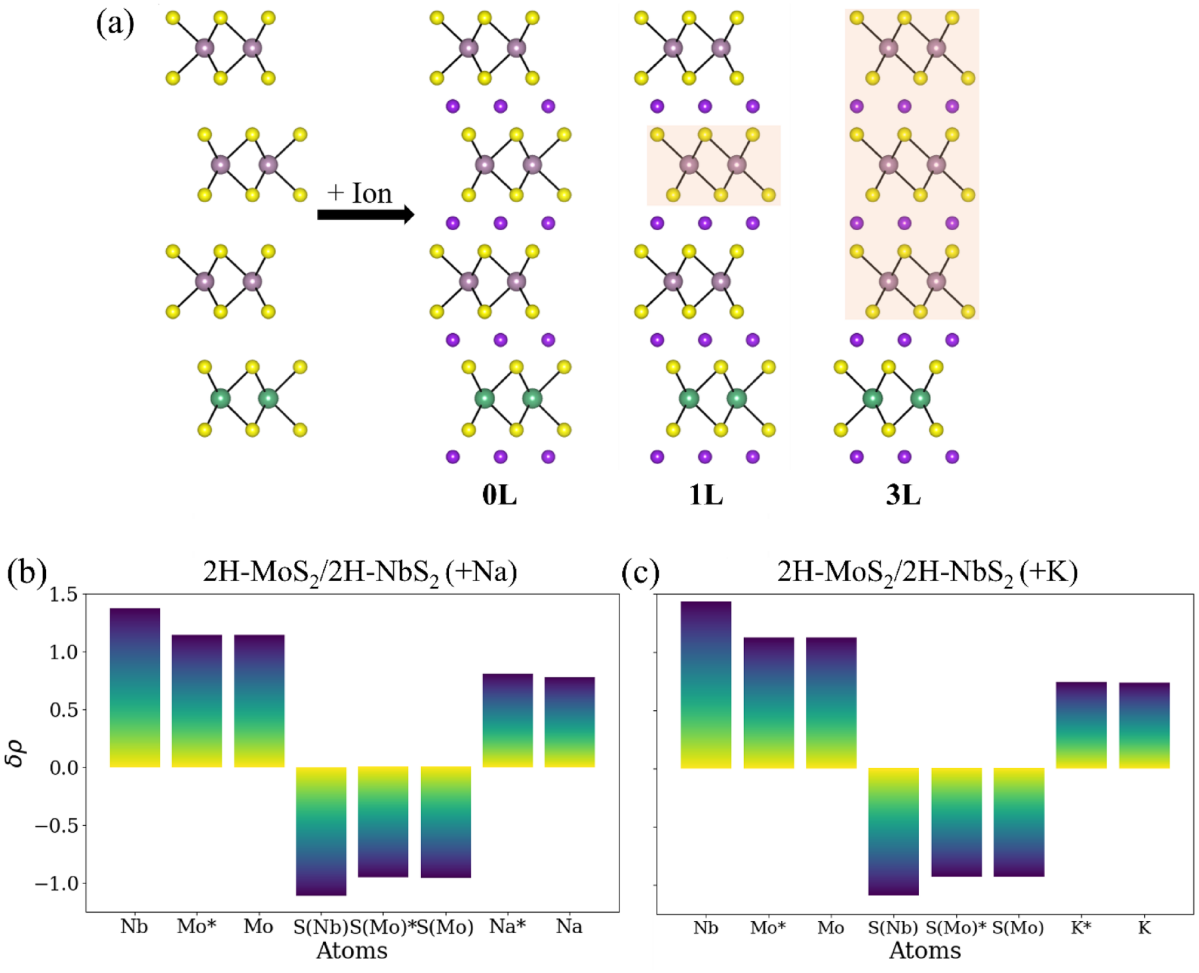
(**a**) (left) Schematic representation of the 3-MoS_2_/1-NbS_2_ heterostructures showing a repeating unit of three MoS_2_ layers and one NbS_2_ layer. Light purple, green, yellow, and darker purple spheres represent Mo, Nb, S, and intercalating ions- Na or K, respectively. 1T-MoS_2_ is highlighted in orange. Charges transferred from different atoms in (**b**) Na-ion intercalated and (**c**) K-ion intercalated lowest energy structure 0L: 2H-NbS_2_/2H-MoS_2_ computed using Bader analysis. A positive value implies that the atom lost electrons, while a negative value indicates that the atom gained electrons. S(Mo) identifies S atoms bonded to Mo, while S(Nb) refers to S atoms bonded to Nb atoms. Asterisks (*) are marked to identify Mo and S(Mo) atoms neighboring NbS_2_ layers and Na- or K-ions intercalating the interface between NbS_2_ and MoS_2_.

It is unclear if this ability to impede the Na-ion and K-ion intercalation-induced phase transformation in the MoS_2_ layer by stacking a layer of NbS_2_ depends on the number of the MoS_2_ layers (thickness of the MoS_2_ film). It remains to be verified if the NbS_2_ layer impedes the phase transformation only in the adjacent MoS_2_ layers or even in layers that are farther away. Hence, multilayered heterostructures are created with a repeating unit of three MoS_2_ layers and one NbS_2_ layer_,_ a structure referred to as 3-MoS_2_/1-NbS_2_, as shown in Fig. 4a. The phase transformation in this multilayered system can occur in several different ways during ion intercalation. Hence three unique configurations of this 3-MoS_2_/1-NbS_2_ heterostructure are created as shown in Fig. 4a: 0L: None of the MoS_2_ layers transform to the 1T phase; 1L: Only one MoS_2_ layer in the middle is transformed to the 1T phase; 3L: All the three MoS_2_ layers are transformed to the 1T phase. The 1L and 3L structures represent the formation of a hybrid 2H/1T phase, as depicted in Fig. 4a. The binding sites for Na- and K-ions during intercalation are investigated using high-symmetry sites to determine minimum energy configurations as tabulated in Note 3 (Table S3) of the Supplemental Information. A comparison of the energies of the Na-ion and K-ion intercalated 3-MoS_2_/1-NbS_2_ structures suggests that the lowest energy configuration is the one where MoS_2_ layers do not phase transform and retain their 2H phase. 2H-MoS_2_ is retained even when MoS_2_ layers are not adjacent to NbS_2_ layers. Thus, in battery anodes, the introduction of NbS_2_ can provide a way to inhibit phase transformation in MoS_2_ during Na-ion and K-ion intercalation. The Bader plots of the 3-MoS_2_/1-NbS_2_heterostructures with three layers of MoS_2_ between two layers on NbS_2_, as shown in Fig. 4b, c, indicate that Na-ions and K-ions intercalated at the interface of MoS_2_ and NbS_2_ layers (marked with *) lose more charge than ions intercalated between two layers of MoS_2_. The difference is more significant during the intercalation of Na-ions than K-ions. The net charge on MoS_2_ layers is slightly more than charges on MoS_2_ in the heterostructure with alternating layers of MoS_2_ and NbS_2_ (difference is 0.02 eV for Na-ion while for K-ions, the charges are same in the two structures)_,_ and hence, even in these structures, phase transformation is not preferred.

**Figure 5:**
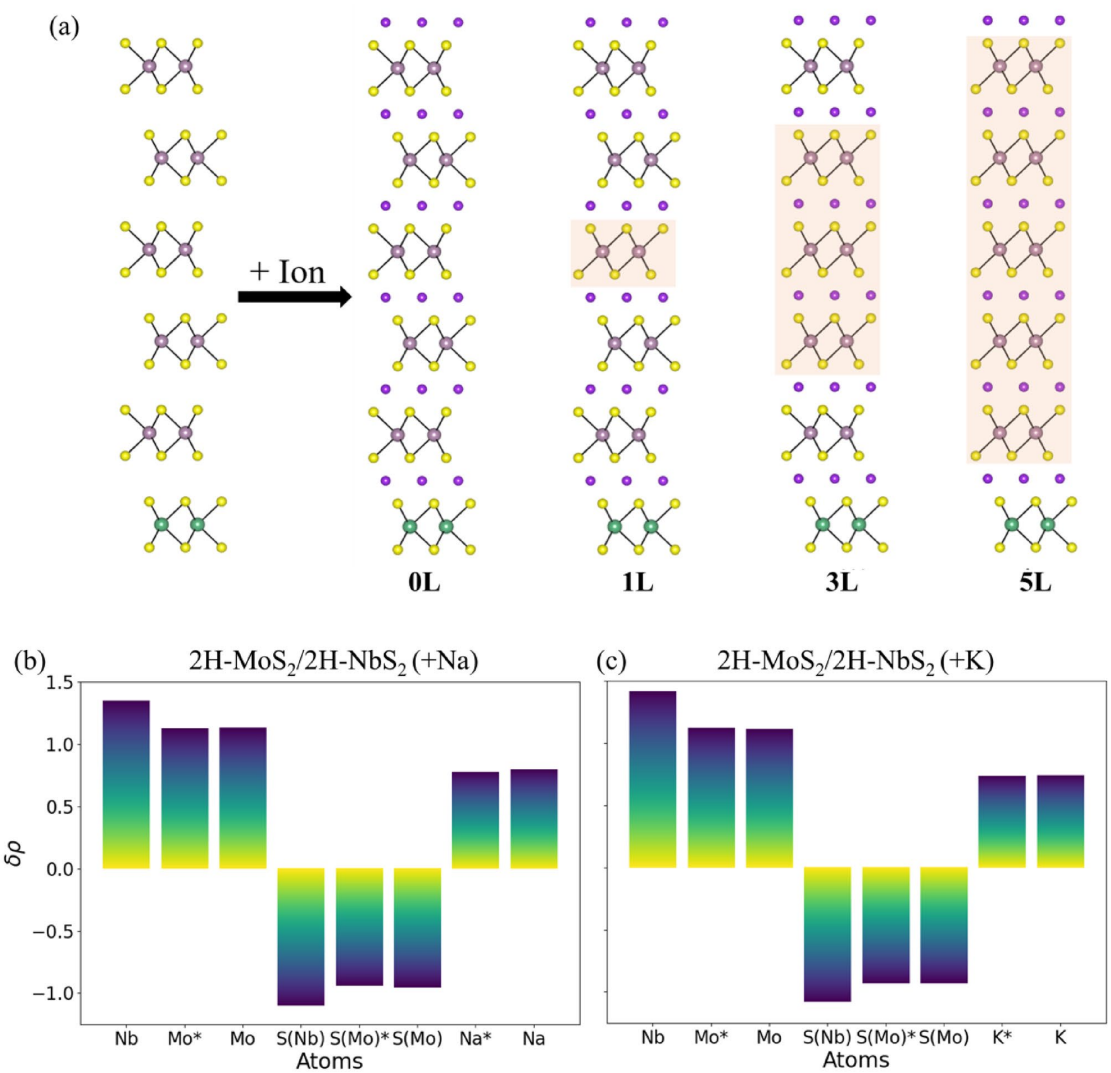
(**a**) (left) Schematic representation of the 5-MoS_2_/1-NbS_2_ heterostructures showing a repeating unit of five MoS_2_ layers and one NbS_2_ layer. Light purple, green, yellow, and darker purple spheres represent Mo, Nb, S, and intercalating ions- Na or K, respectively. 1T-MoS_2_ is highlighted in orange. Charges transferred from different atoms in (**b**) Na-ion intercalated and (**c**) K-ion intercalated lowest energy structure 0L: 2H-NbS_2_/2H-MoS_2_ computed using Bader analysis. A positive value implies that the atom lost electrons, while a negative value indicates that the atom gained electrons. S(Mo) identifies S atoms bonded to Mo, while S(Nb) refers to S atoms bonded to Nb atoms. Asterisks (*) are marked to identify Mo and S(Mo) atoms neighboring NbS_2_ layers and Na- or K-ions intercalating the interface between NbS_2_ and MoS_2_.

DFT calculations are further carried out to investigate the phase stability in multilayered heterostructures created with a repeating unit of five MoS_2_ layers and one NbS_2_ layer_,_ a structure referred to as 5-MoS_2_/1-NbS_2_, as shown in Fig. 5a. The phase transformation in this multilayered system can occur in several different ways during ion intercalation. Hence four unique configurations of this 3-MoS_2_/1-NbS_2_ heterostructure are created as shown in Fig. 5a: 0L: None of the MoS_2_ layers transform to the 1T phase; 1L: Only one MoS_2_ layer in the middle is transformed to the 1T phase; 3L: The three MoS_2_ layers in the middle are transformed to the 1T phase; 5L: The three MoS_2_ layers in the middle are transformed to the 1T phase. The 1L, 3L, and 5L structures represent the formation of a hybrid 2H/1T phase. Bader charges are calculated for Na-ion and K-ion intercalated 5-MoS_2_/1-NbS_2_ heterostructures and these have representes in Fig. 5b, c. The total negative charge on MoS_2_ is same as that seen in Na-ion and K-ion intercalated 3-MoS_2_/1-NbS_2_ heterostructures. The binding sites for Na- and K-ions during intercalation are investigated using high-symmetry sites to determine minimum energy configurations as tabulated in Note 4 (Table S4) of the Supplemental Information. A comparison of the energies of the Na-ion and K-ion intercalated 5-MoS_2_/1-NbS_2_ structures suggests that the lowest energy configuration is the one where MoS_2_ layers do not phase transform and retain their 2H phase. Thus, 2H-MoS_2_ is retained even when MoS_2_ layers are not adjacent to NbS_2_ layers.

DFT simulations are carried out for larger heterostructures by further increasing the number of MoS_2_ layers to seven in the repeating unit with one NbS_2_ layer_;_ a structure referred to as 7-MoS_2_/1-NbS_2_. A comparison of the energies of the Na-ion and K-ion intercalated 7-MoS_2_/1-NbS_2_ structures suggests that the lowest energy configuration is the one where the middle MoS_2_ layer (1L) phase transforms to the 1T phase. Thus, a limit is observed in the capability of one NbS_2_ layer to impede the phase transformation behavior of neighboring MoS_2_ layers during the intercalation of Na-ions than K-ions.

The results suggest that MoS_2_/NbS_2_ heterostructures where every MoS_2_ layer has one NbS_2_ layer as at least the third nearest-neighboring layer are an effective design for layered anodes to retain the phase stability during the intercalation of Na-ions than K-ions. The stability of the phases is attributed to the reduction in the net negative charge on the MoS_2_ layers due to the presence of the NbS_2_ layer during ion intercalation. This difference in charge transfer affects the bond distances between the intercalating ions and NbS_2_ layer and that between the intercalating ions and MoS_2_ layers in the heterostructure as tabulated in Note 5 (Table S5) of the Supplemental Information for the various multilayered heterostructures studied here. This study, therefore, demonstrates the capability of stacking non-transforming TMD layers as an approach to impede the phase transformation of non-adjacent transforming TMD layers in multilayered heterostructures. While this is demonstrated by using NbS_2_ as the non-transforming TMD layers, it is not clear if this strategy will work for other non-transforming TMD layers.

### Intercalation of MoS_2_/VS_2_ multilayered heterostructures

**Figure 6:**
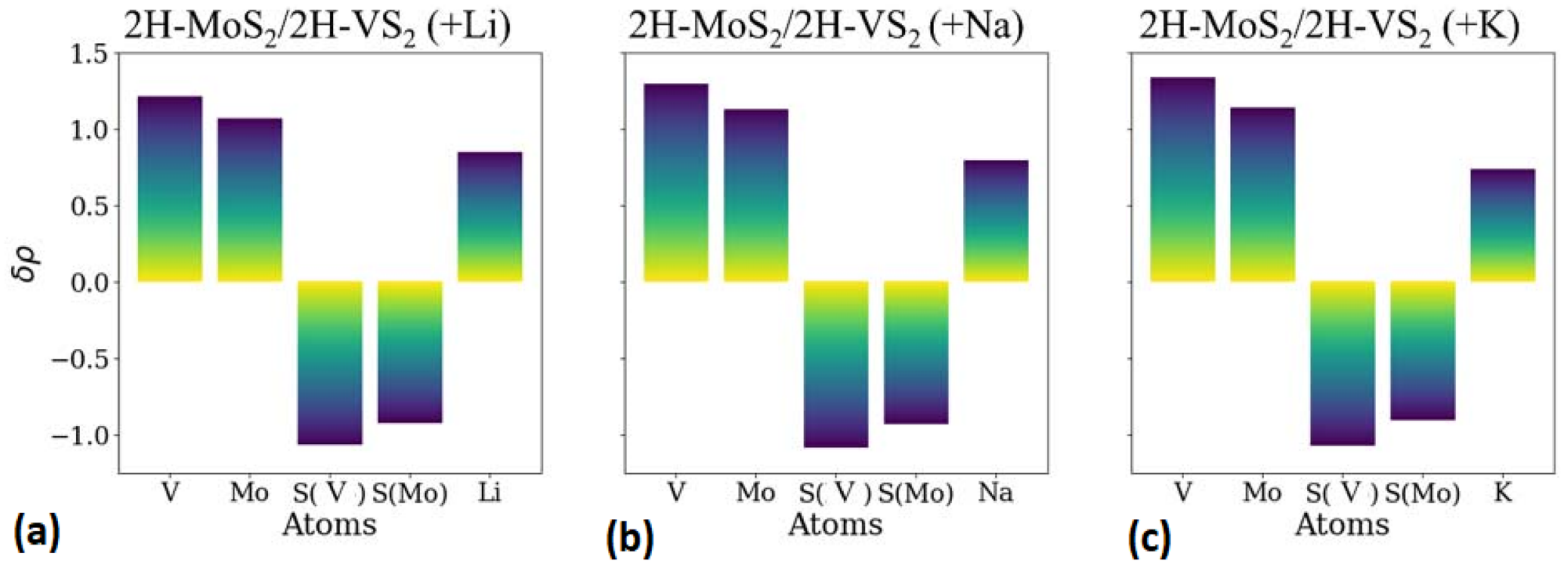
Charges transferred from different atoms in (**a**) Li-ion, (**b**) Na-ion and (**c**) K-ion intercalated 2H-MoS_2_/2H-VS_2_ heterostructures (alternating layers of VS_2_ and MoS_2_) computed using Bader analysis.

DFT simulations are therefore carried out to investigate the phase stability behavior in multilayered heterostructures formed by stacking MoS_2_ layers with VS_2_ layers that do not transform during Li-ion, Na-ion, and K-ion intercalation. *MoS*_*2*_*/VS*_*2*_ heterostructures are first created by stacking one layer of MoS_2_ on top of one layer of VS_2_ to understand the effect of interfaces on the phase transformation behavior of MoS_2_ and VS_2_. This creates a monolayer heterostructure of alternating MoS_2_ and VS_2_ layers and also referred to as the 1-MoS_2_/1-VS_2_ structure. Note 6 (Table S6A) of the Supplemental Information compares the energies of various phases of the MoS_2_ and VS_2_ layers in the intercalated monolayer heterostructure for the possible Na-ion and K-ion binding sites. The total energies of the different phases are compared after Li-ion, Na-ion, and K-ion intercalation, as summarized in Table 3. A comparison of the energies of the Li-ion intercalated (1-MoS_2_/1-NbS_2_) heterostructures suggests that the lowest energy configuration is the one where the MoS_2_ layer does not phase transform and retains the 2H phase for all intercalating ions. This is different from the phase stability of the monolayer MoS_2_/NbS_2_ heterostructures, wherein a phase transformation was observed for Li-ion intercalation. This difference in behavior between NbS_2_ and VS_2_ can be related to their electronic structure and explained based on charge analysis, as shown in Fig. 6a. The net negative charge on MoS_2_ in MoS_2_/VS_2_ heterostructures is lower than the charge on MoS_2_ in MoS_2_/NbS_2_ heterostructures by 0.02 e^-^. This difference can result in impeding the phase transition behavior of the MoS_2_ layer during Li-ion intercalation of 1-MoS_2_/1-VS_2_ heterostructure. Na-ion and K-ion intercalation renders even lower negative charges accumulate on the MoS_2_ layer, as shown in Fig. 6b, c. As a result, no phase transformation is observed during Na-ion and K-ion intercalation. Thus, VS_2_ layers are also able to impede the phase transformation of MoS_2_ layers induced by the intercalation of Li-, Na-, or K-ions.

**Table 3:** Energy of different phases of lithiated 1-MoS_2_/1-VS_2_ heterostructure (with alternating layers of MoS_2_ and VS_2_) after Li, Na and K intercalation.

Ion	2H-MoS_2_/2H-VS_2_ (eV/formula unit)	1T-MoS_2_/2H-VS_2_ (eV/formula unit)	1T-MoS_2_/1T-VS_2_ (eV/formula unit)
Li	**− 50.03**	− 49.96	− 50.00
Na	**− 48.39**	− 48.05	− 48.10
K	**− 47.05**	− 46.68	− 46.84

The numbers in bold identify the lower energy phases of the TMDs in the presence of various intercalating ions.

Similarly, multilayered heterostructures are created with a repeating unit of three MoS_2_ layers and one VS_2_ layer (a structure referred to as 3-MoS_2_/1-VS_2_). Similar to the MoS_2_/NbS_2_ heterostructures, the phase transformation in this multilayered system can occur in several different ways during ion intercalation. Hence three unique configurations of this 3-MoS_2_/1-NbS_2_ heterostructure are created: 0L, 1L, and 3L. The binding sites for Li-, Na-, and K-ions during intercalation are investigated using high-symmetry sites to determine minimum energy configurations as tabulated in Note 6 (Table S6B) of the Supplemental Information. A comparison of the energies of the Na-ion and K-ion intercalated 3-MoS_2_/1-VS_2_ structures suggests that the lowest energy configuration is the one where MoS_2_ layers do not phase transform and retain their 2H phase. However, a comparison of the energetics of the intercalated 3-MoS_2_/1-VS_2_ structures during Li-ion intercalation suggests that the lowest energy configuration is the one where the middle MoS_2_ layer (1L) phase transforms to the 1T phase. The phase transformation barriers of MoS_2_ in Li and Na intercalated heterostructures are 0.9 eV and 0.94 eV, respectively, which is lower than the barriers in bulk MoS_2_. The observed transformation barrier during K intercalation is 0.78 eV in the MoS_2_/VS_2_ heterostructures, which is similar to that seen in the bulk phase.

Thus, 2H-MoS_2_ is retained even when MoS_2_ layers are not adjacent to VS_2_ layers for Na-ion and K-ion intercalation and when the MoS_2_ layer is adjacent to the VS_2_ layer during Li-ion intercalation. The ability to impede the phase transformation behavior of MoS_2_ layers is dependent on the net negative charge rendered on the MoS_2_ layer during ion intercalation.

### Role of charge transfer on phase transformation of multilayered heterostructures

**Figure 7:**
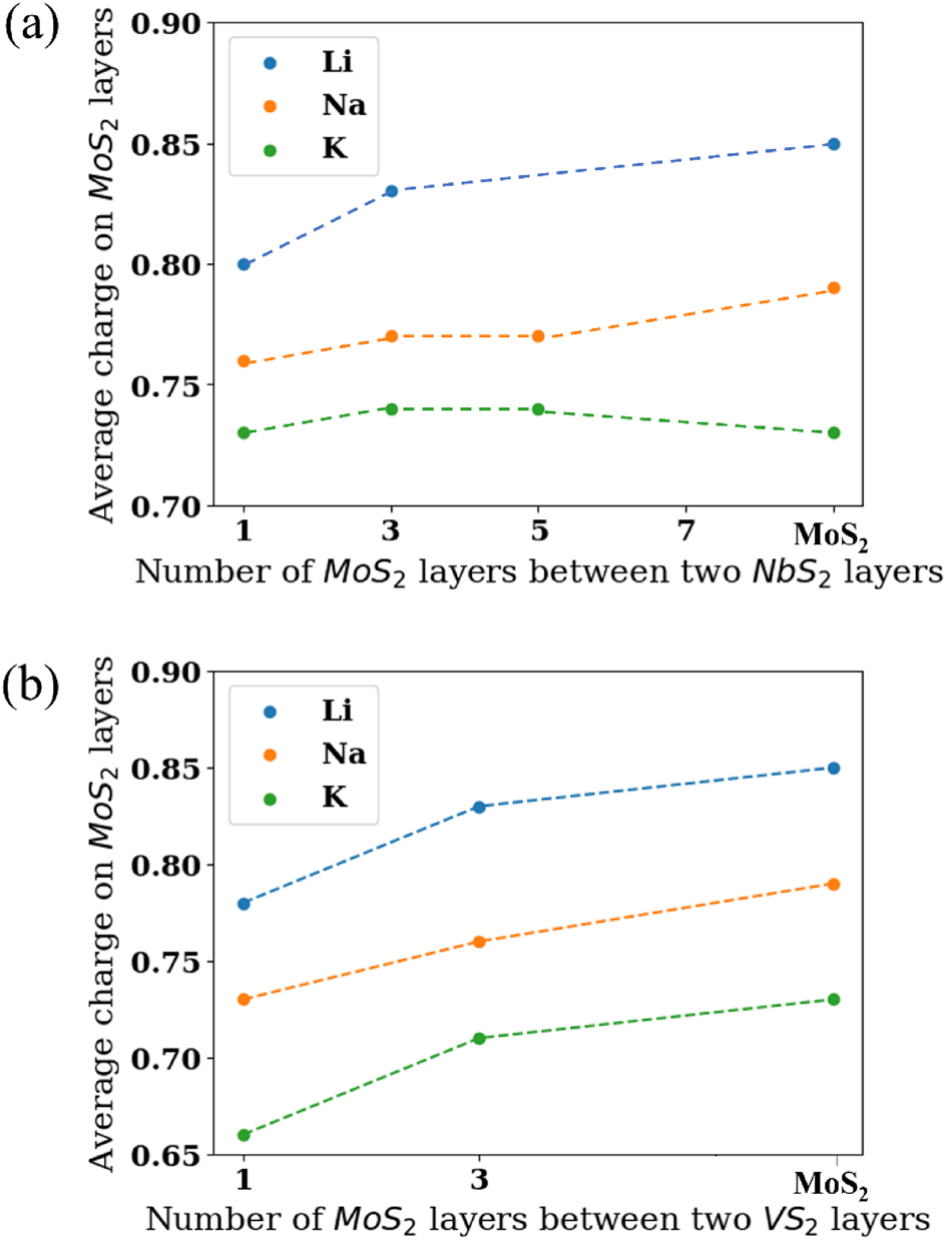
The average negative charge on MoS_2_ layers, upon intercalation with Li-, Na-, and K-ions, as a function of the number of MoS_2_ layers in between two (**a**) NbS_2_ layers and (**b**) VS_2_ layers. The charge on MoS_2_ increases as the number of MoS_2_ layers in between two NbS_2_ (or VS_2_) layers increases.

Throughout this paper, the net charge on MoS_2_ is linked to relative energies of the 2H and 1T phases upon ion intercalation. The net charge on the MoS_2_ layers in intercalated MoS_2_/NbS_2_ and MoS_2_/VS_2_ heterostructures is lower than in the intercalated bulk MoS_2_, as shown graphically in Fig. 7a,b, respectively. As the number of MoS_2_ layers in between the layers of NbS_2_ or VS_2_ increases, the average charge on the MoS_2_ layers also increases. A slight anomaly is seen in the case of K-ion intercalation in MoS_2_/NbS_2_ heterostructures. Upon K-ion intercalation, the net charge on MoS_2_ computed in two of the heterostructures is slightly higher than the charge on MoS_2_ when present in bulk form. This anomaly is most likely due to the precision of the charge calculations since the computed charge values are so close (< 0.01 e^-^). The charge on the MoS_2_ within a given heterostructure is also dependent on how far it is from the NbS_2_ or VS_2_ layers; the MoS_2_ layer closest to the NbS_2_ or VS_2_ layer has the lowest net charge amongst all the layers in the heterostructure. For instance, upon Li-ion intercalation of the MoS_2_/VS_2_ heterostructure where there are three MoS_2_ layers between two layers of VS_2_ (similar to Fig. 4a), the MoS_2_ layers adjacent to VS_2_ layers have a net charge of -0.82 e^-^ while the non-adjacent ones have a net charge of -0.84 e^-^. As discussed in previous sections, the 1T phase is more stable than the 2H phase of MoS_2_ as the charge on the MoS_2_ increases. This hypothesis also holds true in this MoS_2_/VS_2_ microstructure since the most energetically stable configuration is Fig. 4a-1L, where the MoS_2_ layers adjacent to the VS_2_ layers remain in 2H phase while only the non-adjacent layer transforms to 1T phase.

### Capacities and Electrical band gaps of the heterostructures

An important criterion to consider while building such heterostructures is the theoretical capacities as well as the electronic conductivities for the various configurations discussed above. The calculated capacities (Note 7 of Supplemental Information^50^) of 2H-MoS_2_ and the monolayer MoS_2_/NbS_2_ and MoS_2_/VS_2_ heterostructures are summarized in Table 4. The predicted capacity for 2H-MoS_2_ calculated in this study is slightly lower than the reported value of 167 mAh/g, considering a similar anode reaction^51,52^. The difference stems from the fact that previous studies assumed that Li gives up its valance electron completely (n = 1), but the Bader analysis performed in this study shows that Li-, Na-, or K-ions give up partial charges during ion intercalation. Table 4 suggests that MoS_2_/NbS_2_ heterostructures show a small improvement in theoretical capacities over bulk MoS_2_. MoS_2_/VS_2_ heterostructures show much higher capacities giving a > 15% increase of predicted capacities over bulk MoS_2_. A significant improvement of theoretical capacities is expected since the charge transfers from the intercalating ions to the layered materials are similar in bulk MoS_2_ and the heterostructures, but the molecular mass of V is much lower than Nb, which is in turn lower than Mo. The reduction of the weight of the MoS_2_-based anode materials due to the introduction of NbS_2_ or VS_2_ layers can be expected to improve their gravimetric capacities.

**Table 4:** Predicted theoretical capacities of 2H-MoS_2_ and heterostructures with alternating layers of MoS_2_ and NbS_2_ and MoS_2_ and VS_2_ during Li, Na and K intercalation.

Intercalating ion	Layered material	Predicted capacity (mAh/g)
Li	2H-MoS_2_	141.8
	2H-MoS_2_/2H-NbS_2_ (alternating layers)	144.1
	2H-MoS_2_/2H-VS_2_ (alternating layers)	165.5
Na	2H-MoS_2_	131.6
	2H-MoS_2_/2H-NbS_2_ (alternating layers)	136.2
	2H-MoS_2_/2H-VS_2_ (alternating layers)	155.0
K	2H-MoS_2_	121.6
	2H-MoS_2_/2H-NbS_2_ (alternating layers)	124.9
	2H-MoS_2_/2H-VS_2_ (alternating layers)	142.2

**Figure 8:**
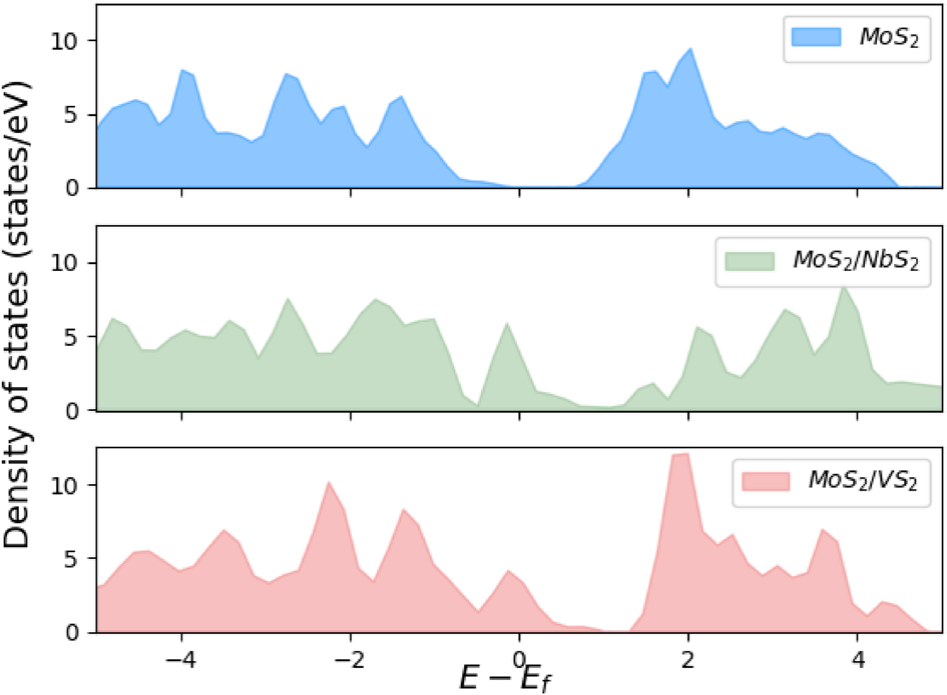
The density of states (DOSs) plots of 2H-MoS_2_ (top), 2H-MoS_2_/2H-NbS_2_ (middle), and 2H-MoS_2_/2H-VS_2_ (bottom). Here, E_f_ denotes the energy of the Fermi level.

The density of states of bulk MoS_2_ and the monolayer MoS_2_/NbS_2_ and the MoS_2_/VS_2_ heterostructures is plotted in Fig. 8. The 2H-MoS_2_ phase is a semiconductor with a wide bandgap close to 0.9 eV for the bulk phase^53^ and hence demonstrates poor electronic conductivities owing to its wide bandgap and can lead to rapid capacity degradation^54^. However, due to the metallic NbS_2_^55^ or VS_2_^56^ layers, the MoS_2_/NbS_2_ and MoS_2_/VS_2_ heterostructures show metallic behavior, which will render significantly higher electrical conductivities than bulk 2H-MoS_2_.

Thus, TMD heterostructures provide an opportunity to tailor the phase transformation behavior of the constituent layers and, at the same time, can result in improved battery performance in terms of capacitance and electrical conductivities. The insights gained from charge transfer calculations suggest that stable heterostructures can be designed by combing individual layers of phase transforming and non-phase transformation TMDs, such that the non-transforming phase exhibits stronger binding with incoming ions. The heterostructures are lighter than bulk MoS_2,_ which leads to significant improvement of the theoretical capacity. In addition to the gain in phase stability and capacities, the addition of metallic NbS_2_ or VS_2_ layers has been shown to decrease the electronic band gap of 2H-MoS_2,_ which can, in turn, result in better electrical conductivity. This study demonstrates a novel method for phase stabilization during ion cycling in batteries by the creation of vdW heterostructures. Similar studies on heterostructures of WS_2_/NbS_2_, WS_2_/VS_2,_ etc., can be performed to validate this claim.

## Conclusion

This paper investigates an approach to impede the phase transformation behavior of TMDs observed during ion intercalation. The approach is based on combining the phase-transforming TMDs with other TMDs that do not phase-transform during ion intercalation to create vdW heterostructures. The model phase-transforming TMD is chosen to be MoS_2_ which transforms from 2H to 1T phase in the intercalation of Li-, Na-, and K-ions, whereas the non-transforming TMD is chosen to be VS_2_ and NbS_2,_ which do not phase transformation during ion intercalation. The phase stability is investigated based on the total energies of the intercalated multilayered heterostructures for various configurations of the number of MoS_2_ layers. The study explores the chemical origin of the phase transformation in MoS_2_ by scrutinizing the charge transfer and binding characteristics. DFT simulations suggest that the presence of an NbS_2_ layer impedes the intercalation-induced 2H→1T phase transformation in MoS_2_ layers that are adjacent to the NbS_2_ layer as well as up to the third-nearest neighbor layer from the NbS_2_ layer for Na-ion and K-ion intercalation. However, the NbS_2_ layer is unable to impede this phase transformation behavior for Li-ion intercalation, even for monolayer heterostructures. Similarly, the presence of a VS_2_ layer impedes the MoS_2_ phase transformation behavior during Li-ion, Na-ion, and K-ion intercalation. The ability to impede the phase transformation behavior of MoS_2_ layers is dependent on the net negative charge rendered on the MoS_2_ layer during ion intercalation. This study demonstrates that TMD heterostructures provide an opportunity to tailor the phase transformation behavior of the constituent layers and, at the same time, can result in improved battery performance in terms of capacitance and electrical conductivities. Such computational studies can also be useful in testing the viability of building heterostructures of other 2D materials, such as layered olivines, spinels, MXenes, etc.

## Methods

### Density functional theory

The first‐principles calculations are carried out within the framework of density functional theory (DFT) using the Vienna Ab‐initio Simulation Package (VASP)^57^. The wave function is represented by a plane‐wave basis with an energy cut‐off of 500 eV. Ion‐electron interactions are expressed by the projector augmented‐wave (PAW)^58^ method, and the generalized gradient approximation formulated by Perdew‐Burke‐Ernzerhof (GGA‐PBE)^59^ is used to express electronic exchange correlations. Periodic boundary conditions are applied so that the unit cell repeats in all directions. The structural parameters for the cell are optimized using the conjugate gradient scheme, and the convergence threshold for residual forces on each atom is set to be 0.01 eV/Å. The DFT-D3 method of Grimme with Becke-Jonson damping^60^ is used to model the vdW interaction between layers in MoS_2_/NbS_2_ systems as it represents well both the relative stability of phases and the structural parameters. DFT-D3 has been extensively used in the literature to model multilayer NbS_2_ and other TMDs^61–63^. For studying such effects in MoS_2_/VS_2_ heterostructures, the DFT-D3 method of Grimme with zero damping has been used to add vdW corrections. This method reproduced structural parameters and phase stabilities of different VS_2_ phases well and has, thus, been also used in previous DFT studies^64^.

## Supplementary Information

Supplementary Information.

## Data Availability

The datasets used and/or analysed during the current study available from the corresponding author on reasonable request.
