# Supplementary material for: Phase engineering of layered anode materials during ion-intercalation in Van der Waal heterostructures
Source: Sci Rep. 2023 Apr 3;13:5408. doi: 10.1038/s41598-023-31342-z (PMC10070316; doi:10.1038/s41598-023-31342-z)
Supplement: Supplementary file 1 — Supplementary Information. [file 41598_2023_31342_MOESM1_ESM.docx]

Supplementary information:

Phase Engineering of Layered Anode Materials during Ion-intercalation in Van der Waal Heterostructures

Shayani Parida^1^, Arthur Dobley^2^, C Barry Carter^3,4^, Avinash M Dongare^1^

# Affiliations

^1^ Department of Materials Science and Engineering, University of Connecticut, Storrs, CT, USA

^2^ Eagle Pitcher Technologies, Providence, RI, USA

^3^ Department of Chemical & Biomolecular Engineering, University of Connecticut, Storrs, CT, USA

^4^ Center for Integrated Nanotechnologies (CINT), Sandia National Laboratories, Albuquerque, NM, USA

*Email: [dongare@uconn.edu](mailto:dongare@uconn.edu)

**Note1: Phase stability of TMDs and binding sites of intercalating ions**

**Table S1A.** The energy of 2H and 1T phases of MoS_2_ intercalated with different ions

| Intercalating ion | Binding site | 2H-MoS_2_  (eV/formula unit) | 1T-MoS_2_  (eV/formula unit) |
| --- | --- | --- | --- |
| Li | O_h_ | -25.83 | **-26.06** |
|  | T_d_ | -25.76 | -25.60 |
| Na | O_h_ | -24.95 | **-25.03** |
|  | T_d_ | -24.87 | -24.57 |
| K | O_h_ | -24.30 | **-24.39** |
|  | T_d_ | -23.94 | -23.88 |
| Mg | O_h_ | -24.51 | **-25.05** |
|  | T_d_ | -24.59 | -24.49 |
| Ca | O_h_ | -25.98 | **-26.50** |
|  | T_d_ | -25.41 | -25.56 |
| Al | O_h_ | -26.27 | -26.05 |
|  | T_d_ | **-26.38** | -26.05 |

**Table S1B.** The energy of 2H and 1T phases of NbS_2_ intercalated with different ions

| Intercalating ion | Binding site | 2H-NbS_2_  (eV/formula unit) | 1T-NbS_2_  (eV/formula unit) |
| --- | --- | --- | --- |
| Li | O_h_ | **-27.05** | -26.61 |
|  | T_d_ | -26.69 | -26.13 |
| Na | O_h_ | **-26.14** | -25.59 |
|  | T_d_ | -25.62 | -25.10 |
| K | O_h_ | **-25.64** | -25.05 |
|  | T_d_ | -25.02 | -24.58 |
| Mg | O_h_ | -25.45 | **-25.61** |
|  | T_d_ | -25.20 | -24.89 |
| Ca | O_h_ | -26.91 | **-27.08** |
|  | T_d_ | -26.38 | -26.21 |
| Al | O_h_ | -26.35 | -26.40 |
|  | T_d_ | **-26.58** | -26.36 |

**Table S1C.** The energy of 2H and 1T phases of VS_2_ intercalated with different ions

| Intercalating ion | Binding site | 2H-VS_2_  (eV/formula unit) | 1T-VS_2_  (eV/formula unit) |
| --- | --- | --- | --- |
| Li | O_h_ | **-24.78** | -24.50 |
|  | T_d_ | -24.46 | -24.10 |
| Na | O_h_ | **-23.96** | -23.57 |
|  | T_d_ | -23.43 | -23.16 |
| K | O_h_ | **-23.25** | -22.96 |
|  | T_d_ | -22.70 | -22.47 |
| Mg | O_h_ | -23.13 | **-23.47** |
|  | T_d_ | -22.91 | -22.70 |
| Ca | O_h_ | -24.56 | **-24.89** |
|  | T_d_ | -23.85 | -24.00 |
| Al | O_h_ | -24.15 | -24.18 |
|  | T_d_ | **-24.48** | -24.26 |

**Note 2: Intercalation of 1-MoS_2_/1-NbS_2_ heterostructures with Li-, Na-, and K-ions**

Figure S1. Pictorial representation of NbS_2_/MoS_2_ heterostructure with alternating MoS_2_ and NbS_2_ layers. The green, purple, and yellow spheres represent Nb, Mo, and S atoms.

**Table S2A.** Energetics of different phases of MoS_2_/NbS_2_ monolayer heterostructure (alternating layers of MoS_2_ and NbS_2_).

|  | 2H-MoS_2_/2H-NbS_2_ | 1T-MoS_2_/2H-NbS_2_ | 2H-MoS_2_/1T-NbS_2_ | 1T-MoS_2_/1T-NbS_2_ |
| --- | --- | --- | --- | --- |
| Energy (eV)/ formula unit | -45.56 | -44.85 | -45.46 | -44.74 |

**Table S2B.** The energy of different phases of lithiated 1-MoS_2_/1-NbS_2_ monolayer heterostructure (with alternating layers of MoS_2_ and NbS_2_) upon Li-ion, Na-ion and K-ion intercalation

| Ion | Binding site | 2H-MoS_2_/2H-NbS_2_  (eV/formula unit) | 1T-MoS_2_/2H-NbS_2_  (eV/formula unit) | 1T-MoS_2_/1T-NbS_2_ (eV/formula unit) |
| --- | --- | --- | --- | --- |
| Li | H site | -52.75 | -52.56 | -51.69 |
|  | Top Mo site | -52.06 | -52.42 | -52.64 |
|  | Top Nb site | -52.57 | **-52.76** | - |
| Na | H site | **-50.96** | -50.62 | -49.62 |
|  | Top Mo site | -50.02 | -50.17 | -50.56 |
|  | Top Nb site | -50.39 | -50.66 | - |
| K | H site | **-49.93** | -49.49 | -48.45 |
|  | Top Mo site | -48.72 | -48.94 | -49.43 |
|  | Top Nb site | -49.10 | -49.48 | - |

**Note 3: Intercalation of 3-MoS_2_/1-NbS_2_ heterostructures with Na-, and K-ions**

**Table S3.** The energy of different phases of lithiated 3-MoS_2_/1-NbS_2_ heterostructure a repeating unit of three MoS_2_ layers and one NbS_2_ layer upon Na and K-ions intercalation at various binding sites.

| Ion | Site | 3-MoS_2_/1-NbS_2_  0L  (eV/formula unit) | 3-MoS_2_/1-NbS_2_  1L  (eV/formula unit) | 3-MoS_2_/1-NbS_2_  3L  (eV/formula unit) |
| --- | --- | --- | --- | --- |
| Na | H | **-100.79** | -100.42 | -99.72 |
|  | Top Mo | -99.38 | -99.53 | -100.19 |
|  | Top Nb | -99.79 | -99.86 | -99.76 |
| K | H | **-98.52** | -98.13 | -97.31 |
|  | Top Mo | -96.58 | -96.90 | -97.73 |
|  | Top Nb | -96.98 | -97.18 | -97.33 |

**Note 4: Intercalation of 5-MoS_2_/1-NbS_2_ heterostructures with Na-, and K-ions**

**Table S4.** The energy of different phases of lithiated 5-MoS_2_/1-NbS_2_ heterostructure a repeating unit of three MoS_2_ layers and one NbS_2_ layer upon Na and K-ions intercalation at various binding sites.

| Ion | Site | 5-MoS_2_/1-NbS_2_  0L  (eV/formula unit) | 5-MoS_2_/1-NbS_2_  1L  (eV/formula unit) | 5-MoS_2_/1-NbS_2_  3L  (eV/formula unit) | 5-MoS_2_/1-NbS_2_  5L  (eV/formula unit) |
| --- | --- | --- | --- | --- | --- |
| Na | H | **-150.67** | -150.30 | -149.54 | -148.87 |
|  | Top Mo | -148.86 | -148.93 | -150.10 | -150.28 |
|  | Top Nb | -149.27 | -149.38 | -148.53 | -148.91 |
| K | H | **-147.11** | -146.67 | -145.87 | -145.11 |
|  | Top Mo | -144.44 | -144.60 | -146.32 | -146.59 |
|  | Top Nb | -144.84 | -145.21 | -144.40 | -145.10 |

**Note 5: Bonding in multilayered heterostructures during intercalation of Li-, Na-, and K-ions**

**Table S5.** Interatomic distances in MoS_2_ and MoS_2_-NbS_2_ heterostructures. S(Mo) and S(Nb) denote S atoms attached to Mo and Nb, respectively. Here, ‘*’ is used to denote atoms present on the interface of NbS_2_ and MoS_2_ layers in the heterostructures.

| Ion | Bond type | MoS_2_ | 1-MoS_2_/1-NbS_2_  (Å) | 3-MoS_2­_/1-NbS_2_ (Å) | 5-MoS_2­_/1-NbS_2_  (Å) |
| --- | --- | --- | --- | --- | --- |
| Li | Mo - Mo | 7.01 | - | 6.96 | - |
|  | Mo - Nb | - | 6.76 | 6.82 | - |
|  | S(Mo) - Li | 2.52 | - | 2.53 | - |
|  | S(Mo)* - Li | - | 2.56 | 2.55 | - |
|  | Mo - S | 2.45 | - | 2.46 | - |
|  | Mo* - S | - | 2.46 | 2.44 | - |
|  | S(Nb) - Li | - | 2.45 | 2.44 | - |
| Na | Mo - Mo | 7.67 | - | 7.53 | 7.55 |
|  | Mo - Nb | - | 7.37 | 7.38 | 7.45 |
|  | S(Mo) – Na | 2.82 | - | 2.82 | 2.82 |
|  | S(Mo)* - Na | - | 2.81 | 2.81 | 2.82 |
|  | Mo - S | 2.45 | - | 2.45 | 2.46 |
|  | Mo* - S | - | 2.46 | 2.44 | 2.44 |
|  | S(Nb) - Na | - | 2.73 | 2.72 | 2.73 |
| K | Mo - Mo | 7.62 | - | 7.63 | 7.63 |
|  | Mo - Nb | - | 7.76 | 7.75 | 7.74 |
|  | S(Mo) – K | 3.05 | - | 3.05 | 3.05 |
|  | S(Mo)* - K | - | 3.06 | 3.06 | 3.05 |
|  | Mo - S | 2.46 | - | 2.45 | 2.45 |
|  | Mo* - S | - | 2.45 | 2.45 | 2.45 |
|  | S(Nb) - K | - | 3.04 | 3.05 | 3.05 |

**Note 6: Intercalation of 1-MoS_2_/1-VS_2_ heterostructures with Li-, Na-, and K-ions**

**Table S6A.** Energy of different phases of intercalated MoS_2_/VS_2_ heterostructure (with alternating layers of MoS_2_ and VS_2_) upon Li-ion, Na-ion, and K-ion intercalation

| Intercalating ion | Binding site | 2H-MoS_2_/2H-VS_2_  (eV/formula unit) | 1T-MoS_2_/2H-VS_2_  (eV/formula unit) | 1T-MoS_2_/1T-VS_2_  (eV/formula unit) |
| --- | --- | --- | --- | --- |
| Li | H | **-50.03** | -49.81 | -49.17 |
|  | Top Mo | -49.38 | -49.68 | -50.00 |
|  | Top V | -49.93 | -49.96 | - |
| Na | H | **-48.39** | -48.05 | -47.25 |
|  | Top Mo | -47.45 | -47.53 | -48.10 |
|  | Top V | -47.89 | -48.03 | - |
| K | H | **-47.05** | -46.68 | -45.95 |
|  | Top Mo | -45.91 | -46.09 | -46.84 |
|  | Top V | -46.35 | -46.63 | - |

**Table S6B.** The energy of different phases of intercalated 3-MoS_2_/1-VS_2_ heterostructure a repeating unit of three MoS_2_ layers and one VS_2_ layer upon Na and K-ions intercalation at various binding sites.

| Ion | Site | 3-MoS_2_/1-VS_2_  0L  (eV/formula unit) | 3-MoS_2_/1-VS_2_  1L  (eV/formula unit) | 3-MoS_2_/1-VS_2_  3L  (eV/formula unit) |
| --- | --- | --- | --- | --- |
| Li | H | -100.52 | -100.31 | -99.95 |
|  | Top Mo | -99.83 | -99.83 | -100.66 |
|  | Top V | -100.37 | **-100.69** | -100.10 |
| Na | H | **-97.33** | -96.99 | -96.32 |
|  | Top Mo | -96.01 | -96.08 | -96.61 |
|  | Top V | -96.44 | -96.48 | -96.30 |
| K | H | **-94.69** | -94.33 | -93.61 |
|  | Top Mo | -92.91 | -93.21 | -93.93 |
|  | Top V | -93.41 | -93.49 | -93.60 |

**Note 7: Calculation of theoretical capacities of heterostructures**

The theoretical capacity of a material is determined using the equation^1^:

$Q_{theoretical}=\frac{n\times F}{3600\times M}$*.* Here,$n is the number of electrons exchanged between the ion and the 2D material$, $F is the Faraday constant (96485.3329\frac{\mathrm{sA}}{\mathrm{mol}})$

and $M is the molecular weight of the 2D material$.

To compare the capacities of the heterostructures with the clean TMDs, fully intercalated configurations are considered wherein the ions occupy all the octahedral sites. The anode, M_x_Mo_(1–x)_S_2_ (M refers to V or Nb atoms), in such a scenario would undergo the reaction^2^:

*M_x_Mo_(1-x)_S_2_ + Li^n+^ + ne^−^ → LiM_x_Mo_(1-x)_S_2_* (2)

Here, 0 ≤ x ≤ 1

# **References**

1 He, Q., Yu, B., Li, Z. & Zhao, Y. Density Functional Theory for Battery Materials. *Energy Environ. Sci.* **2**, 264-279, doi:<https://doi.org/10.1002/eem2.12056> (2019).

2 Stephenson, T., Li, Z., Olsen, B. & Mitlin, D. Lithium ion battery applications of molybdenum disulfide (MoS_2_) nanocomposites. *Energy Environ. Sci.* **7**, 209-231, doi:10.1039/C3EE42591F (2014).
